# Rheumatoid Arthritis-Associated Interstitial Lung Disease Mortality Trends in the United States: A Population-Based Analysis (1999–2020)

**DOI:** 10.31138/mjr.061225.ldt

**Published:** 2026-06-12

**Authors:** Farheen Malik, Jawad Ahmed, Mandar Shah, Ritika Uttam, Muhammad Fahimuddin

**Affiliations:** 1 Internal Medicine Residency Program, Jacobi Medical Centre - Albert Einstein College of Medicine, Bronx, NY, USA;; 2 Department of Medicine, Northwest Health Porter, Valparaiso, IN, USA;; 3 Department of Medicine, Jacobi Medical Centre - Albert Einstein College of Medicine, Bronx, NY, USA

**Keywords:** rheumatoid arthritis, interstitial lung disease, mortality trends, epidemiology, CDC WONDER, health disparities

## Abstract

**Objective:**

To evaluate long term trends and demographic, racial, and geographic disparities in mortality related to rheumatoid arthritis associated interstitial lung disease (RA-ILD) in the United States from 1999 to 2020.

**Methods:**

Mortality data were taken from the CDC WONDER Multiple Cause of Death database for individuals aged 15 and older. Deaths were included if both ICD-10 codes for rheumatoid arthritis (M05–M06) and interstitial lung disease with fibrosis (J84.1) were recorded; juvenile RA (M08.0) was excluded. Age-adjusted mortality rates (AAMRs) per 100,000 individuals were computed using the 2000 U.S. standard population. Trends were analysed through average and segmented annual percent changes (AAPC, APC) by sex, age, race/ethnicity, geography, and rural urban classification.

**Results:**

Between 1999 and 2020, there were 10,171 RA-ILD related deaths. The overall AAMR was 0.2 per 100,000, declining from 0.2 in 1999 to 0.1 in 2020 (AAPC –3.08; 95% CI –4.79 to –2.28; p<0.01). Females represented 63.5% of deaths, exhibiting higher AAMRs than males (0.2 versus 0.1). American Indian/Alaska Native individuals had the highest mortality rate (0.5), while non-Hispanic Black and Asian/Pacific Islander groups had the lowest (0.1). The most significant decline occurred from 2014 to 2017 (APC –24.09; p<0.05). Geographic disparities persisted, especially in northern and western states.

**Conclusion:**

Mortality from RA-ILD has decreased over the past two decades, particularly post 2014, likely due to enhanced management and early detection of ILD. However, ongoing gender, racial, and regional disparities emphasise the necessity for equitable healthcare access.

## INTRODUCTION

Rheumatoid arthritis (RA) is a systemic condition distinguished by the inflammation of joints (arthritis), with an incidence rate of approximately 0.5 to 1.0% in the regions of Europe and North America.^1^ The disease causes persistent synovial inflammation and joint involvement, while also encompassing a wide array of extra-articular manifestations, which include, but are not limited to, rheumatoid nodules and vasculitis, as well as diseases affecting the cardiovascular (CV), pulmonary, neurological, gastrointestinal, renal, and hematologic systems. Numerous research groups have documented a decrease in the incidence of RA during the latter half of the 20th century.^2–4^ Interstitial lung disease (ILD) is a clinically significant pulmonary complication of RA, associated with progressive morbidity and premature mortality.^5^ While improved RA management over the last two decades has transformed patient outcomes, the effect of these advances on RA-ILD mortality has not been fully defined. Earlier studies using national mortality datasets have reported conflicting findings, with some suggesting stable or increasing mortality and others indicating gradual improvement. Given ongoing therapeutic changes, increasing use of biologic and targeted synthetic disease-modifying antirheumatic drugs (DMARDs), and growing recognition of RA-ILD as a distinct clinical entity, it is vital to re-examine long-term mortality patterns at a population level. We therefore aimed to analyse national trends in RA-ILD mortality, stratified by demographic and geographic factors, using the CDC WONDER database.

## METHODS

We analysed mortality data from the CDC WONDER Multiple Cause of Death database, covering the period from January 1999 to December 2020, for individuals aged 15 years and older. Deaths were included if International Classification of Diseases, Tenth Revision (ICD-10) codes for rheumatoid arthritis (RA) and interstitial lung disease with fibrosis (J84.1) were both listed. Juvenile RA (M08.0) was excluded.

RA was defined using ICD-10 codes M05 (seropositive RA: M05.0 Felty syndrome, M05.1 RA with lung disease, M05.2 RA vasculitis, M05.3 RA with other systemic involvement, M05.8 other seropositive RA, M05.9 seropositive RA unspecified) and M06 (other RA: M06.0 seronegative RA, M06.2 RA bursitis, M06.3 RA nodule, M06.8 other specified RA, M06.9 RA unspecified). Age-adjusted mortality rates (AAMR) per 100,000 persons were calculated using the U.S. 2000 standard population. Temporal trends were assessed using average annual percent change (AAPC) and segmented annual percent change (APC), both with 95% confidence intervals. Analyses were stratified by sex, age group, race/ethnicity, geographic region, and rural–urban status. MeSH used to characterise the study included Rheumatoid Arthritis, Interstitial Lung Disease, Pulmonary Fibrosis, Mortality, Epidemiology, Population Surveillance, Health Disparities, Cause of Death, and United States.

This study was reported in accordance with the Strengthening Reporting of Observational Studies in Epidemiology (STROBE) guidelines.

## RESULTS

### Overall Mortality Trends

From 1999 to 2020, 10,171 deaths were identified with both RA and ILD recorded. The overall age-adjusted mortality rate (AAMR) was 0.2 per 100,000, declining from 0.2 in 1999 to 0.1 in 2020 (AAPC –3.08; 95% CI –4.79 to –2.28; p<0.01). Trends showed three phases: a non-significant decline from 1999–2014 (APC –0.17; 95% CI –0.98 to 0.74), a sharp reduction from 2014–2017 (APC –24.09; 95% CI –31.19 to –5.09; p<0.05), and a modest, non-significant increase from 2017–2020 (APC +6.70; 95% CI –10.87 to 36.54).

In a sensitivity analysis excluding ICD-10 codes M05.1 and M05.2, the AAMR remained similar at 0.2 per 100,000 (95% CI: 0.1–0.2), compared to the primary estimate of 0.2 per 100,000. The number of deaths decreased from 10,171 to 9,562, resulting in a slightly wider confidence interval. These findings suggest that the overall mortality estimates were not driven by these specific RA subtypes.

### Age-Specific Mortality

Mortality rose with age. Deaths under 25 years were suppressed (<10), and mortality rates for ages 25–34 (n=11) and 35–44 (n=65) were unreliable. Among those aged 45–54, 325 deaths were recorded with negligible CMR. Higher rates appeared from age 55 onward: 1,225 deaths at ages 55–64 (CMR 0.2), 2,907 at 65–74 (CMR 0.6), 3,951 at 75–84 (CMR 1.3), and 1,684 at ≥85 years (CMR 1.4).

### Geographic Variation

**Figure 1.**
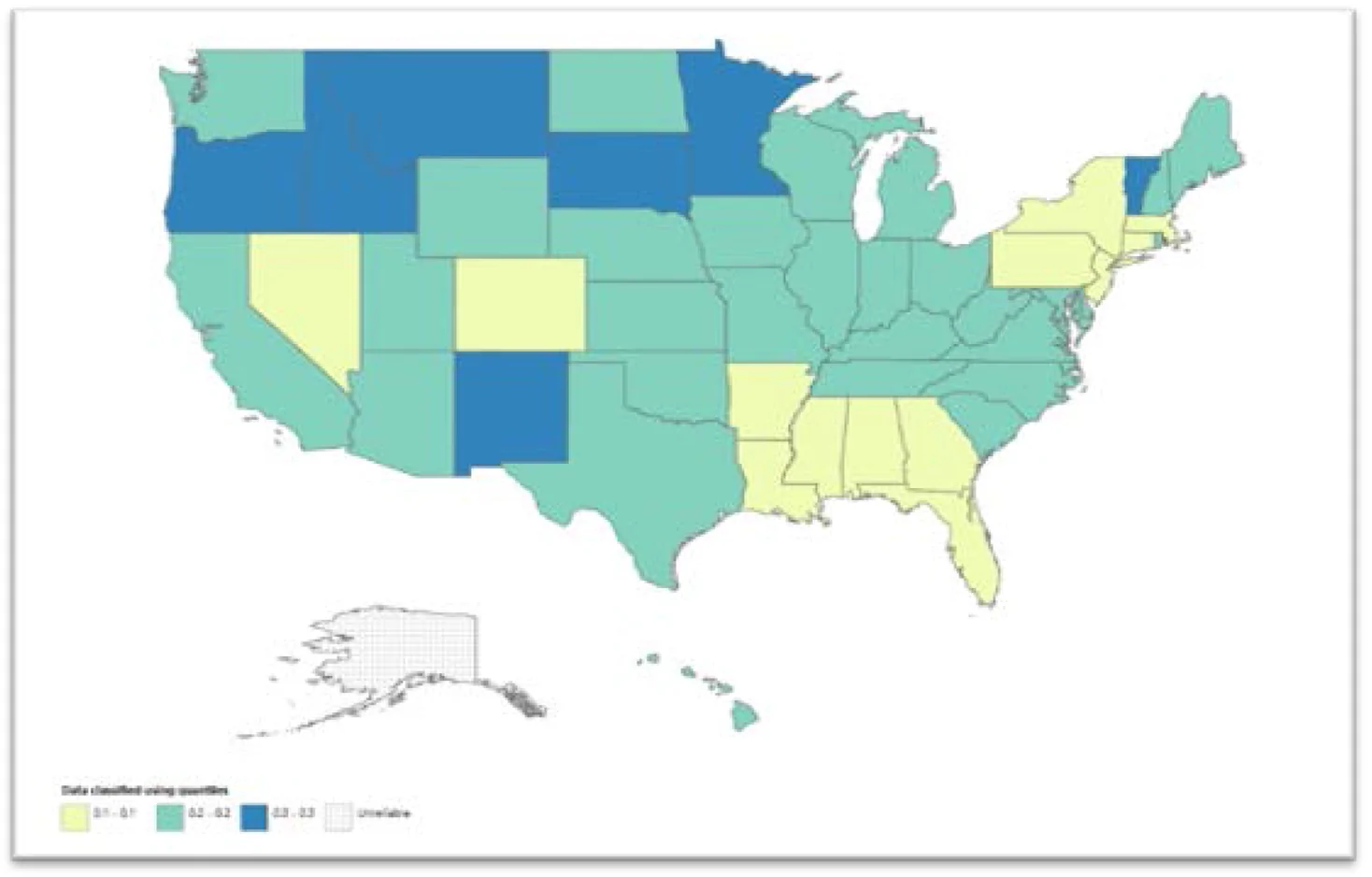
Geographic Distribution of RA-ILD Mortality Rates by State (1999–2020).

**Figure 2.**
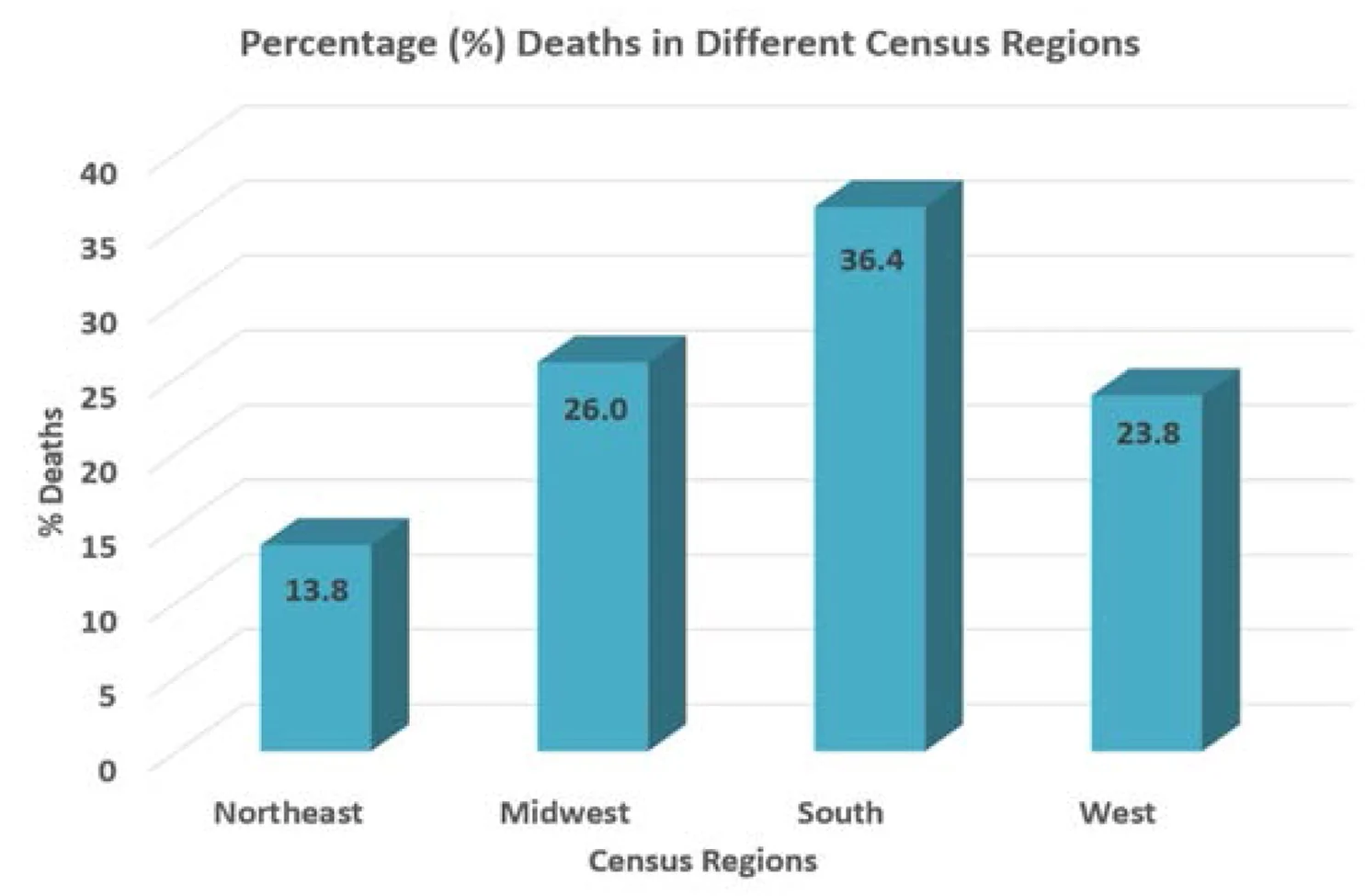
Visual depiction of mortality trends in different census regions.

The Northeast had the lowest AAMR (0.1), whereas the Midwest, South, and West each had 0.2. At the state level, Idaho, Minnesota, Montana, New Mexico, Oregon, South Dakota, and Vermont reported the highest AAMR (**Figures 1** and **2**).

### Racial and Ethnic Disparities

**Figure 3.**
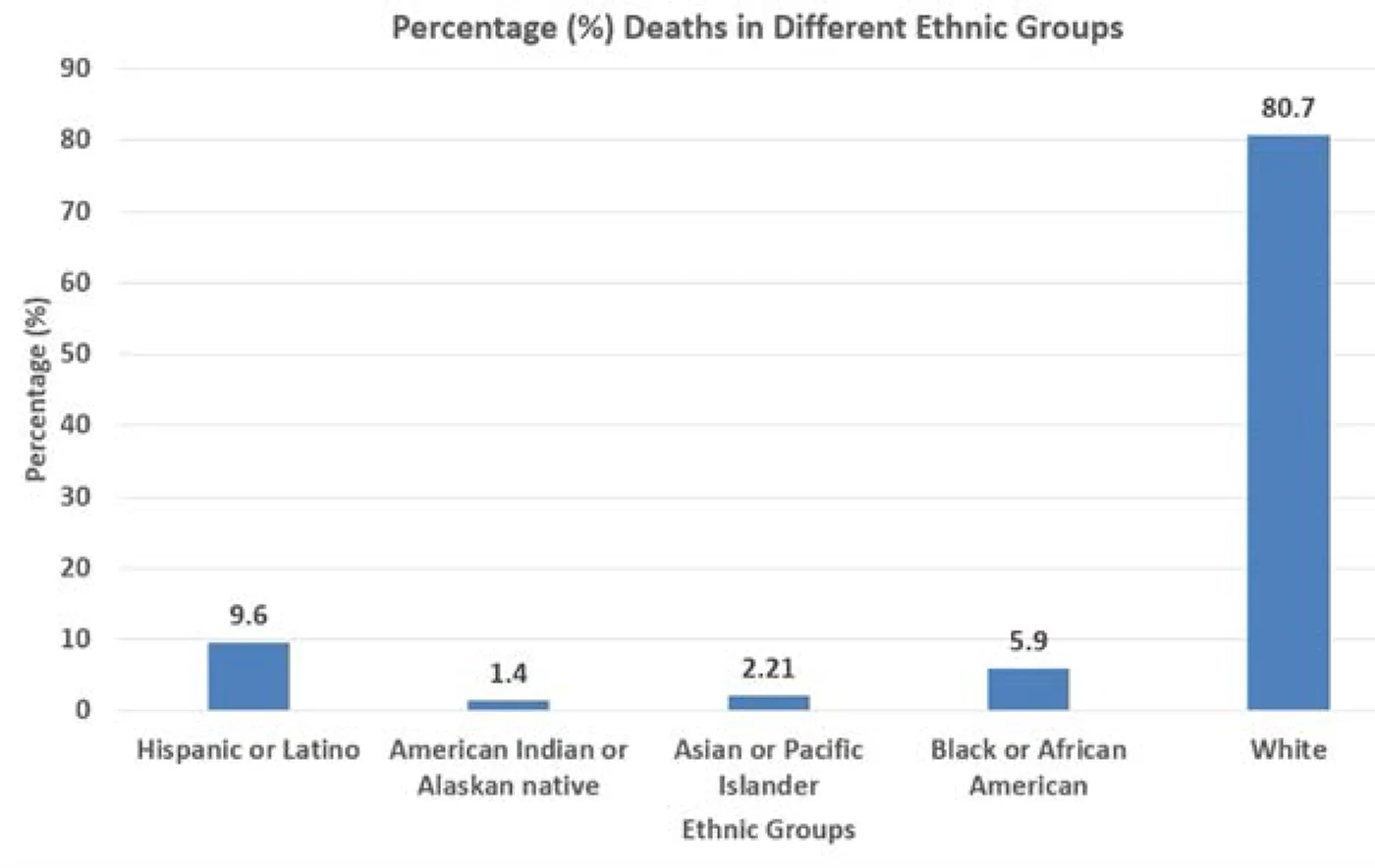
Visual depiction of mortality trends among different ethnic groups.

American Indian/Alaska Native individuals had the highest AAMR (0.5; 95% CI 0.4–0.5), while non-Hispanic Black and Asian/Pacific Islanders had the lowest (0.1; 95% CI 0.1–0.1). Whites comprised 80.7% of deaths (n=8,204) with no significant change from 1999–2014 (APC –0.01), but a significant decline from 2014–2020 (APC –11.99; p<0.01). Hispanics represented 9.6% of deaths (n=980) with a non-significant decline over time (APC –0.81; 95% CI –2.43 to 0.83). African American/Black individuals accounted for 5.9% (n=601) and Asian/Pacific Islanders for 2.2% (n=225) (**Figure 3**).

### Sex Differences

**Figure 4.**
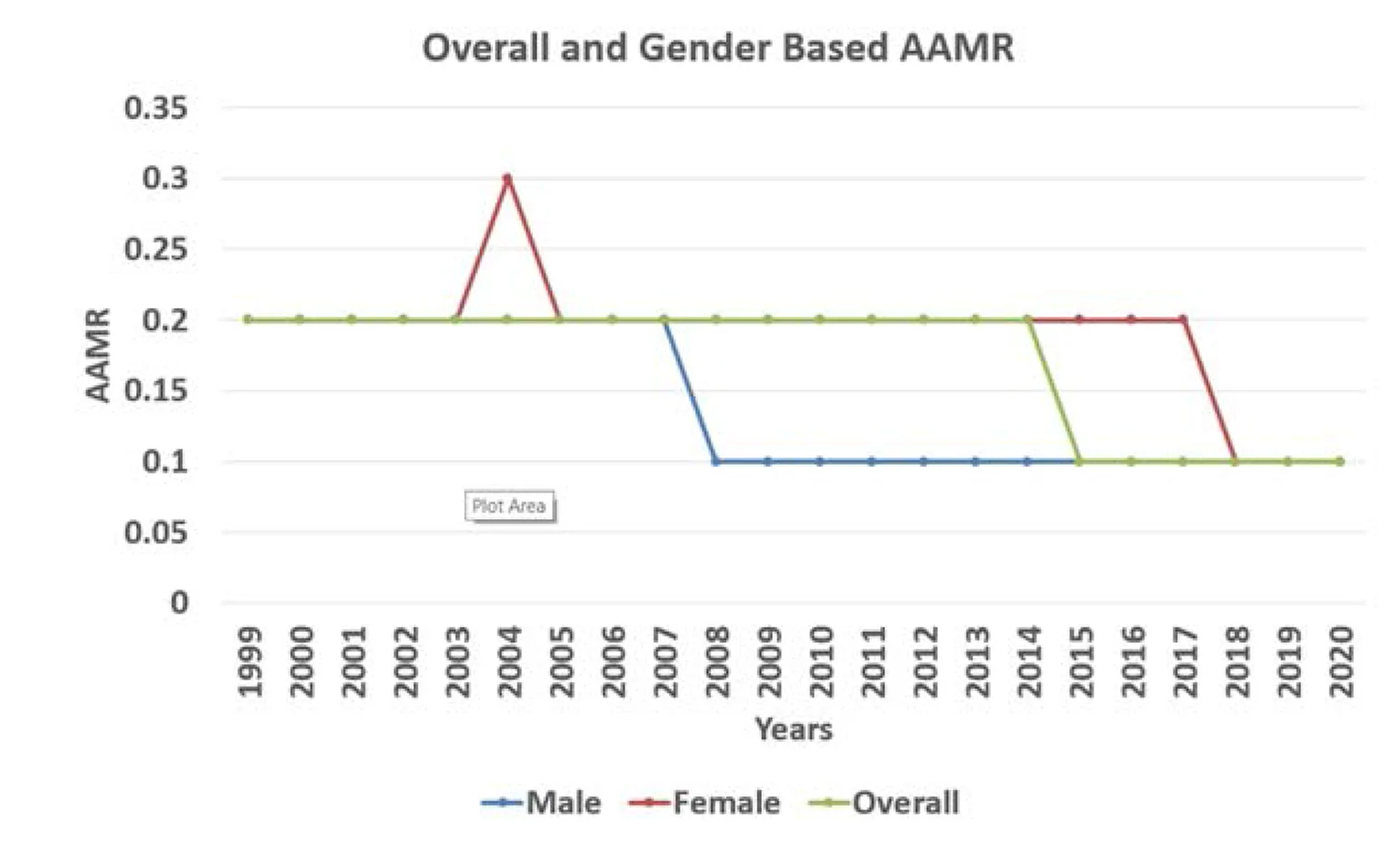
Visual depiction of mortality trends, overall and gender-based.

Women accounted for 63.5% of deaths (n=6,463) and had higher AAMR than men (0.2 vs. 0.1). Women experienced a gradual, non-significant decline from 1999– 2017 (APC –0.69; 95% CI –1.89 to 2.16), followed by a significant drop from 2017–2020 (APC –24.97; 95% CI –43.01 to –2.83). In men, AAMR decreased modestly from 1999–2007 (APC –0.65; 95% CI –2.08 to 1.82), declined sharply from 2007–2010 (APC –21.89; 95% CI –27.05 to –12.4), then rose non-significantly from 2010–2020 (APC +1.14; 95% CI –1.52 to 7.19) (**Figure 4**).

### Rural–Urban Differences

**Figure 5.**
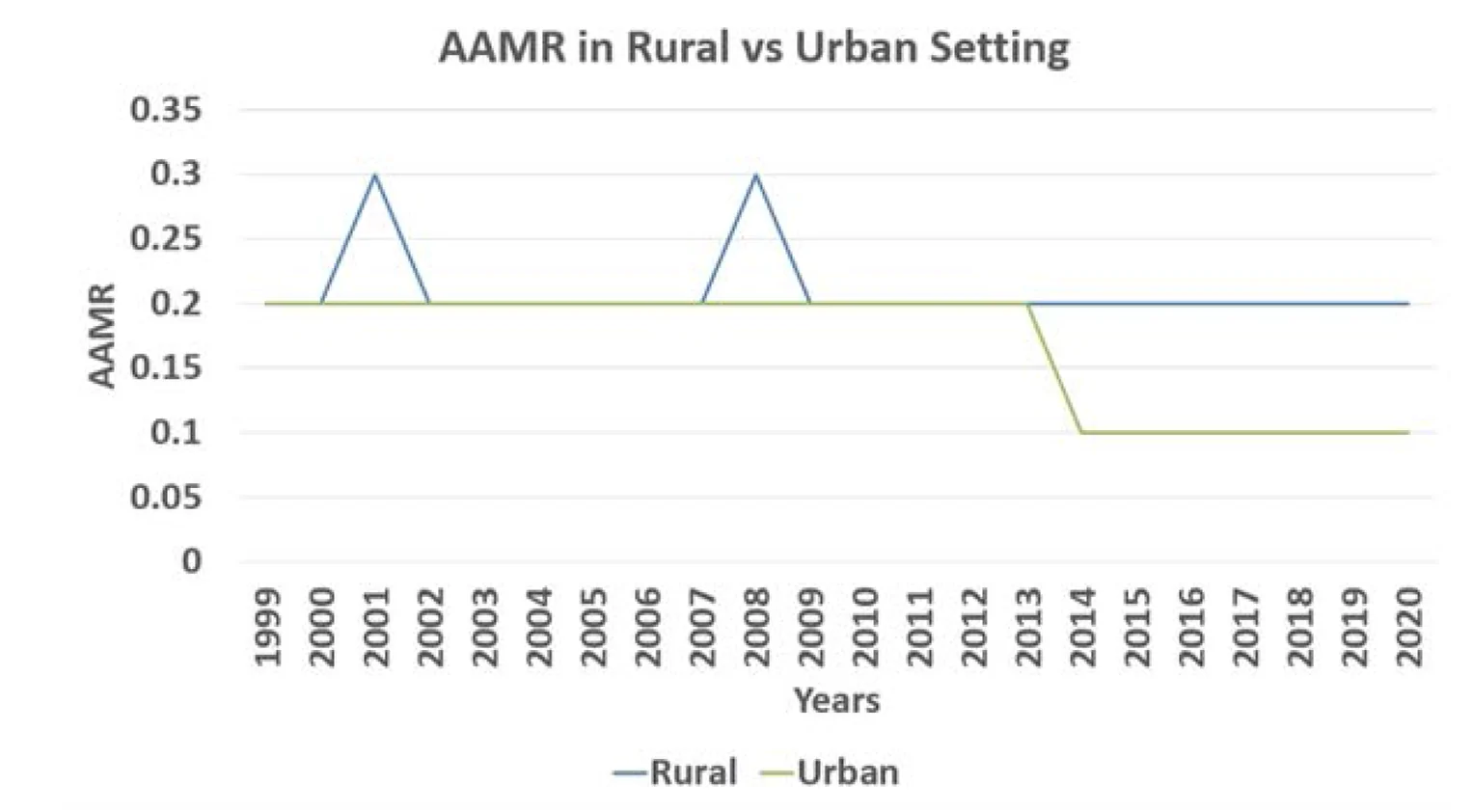
Visual depiction of mortality trends, rural vs urban setting.

AAMR was similar in rural and urban populations (0.2). Rural areas declined non-significantly from 1999–2020 (APC –0.84; 95% CI –1.93 to 0.22). Urban areas declined slightly from 1999–2013 (APC –0.20; 95% CI –0.99 to 0.78), dropped significantly from 2013–2016 (APC –23.64; 95% CI –30.21 to –14.88; p<0.01), and rose non-significantly from 2016–2020 (APC +4.60; 95% CI –5.68 to 34.15) (**Figure 5**).

In a sensitivity analysis excluding ICD-10 codes M05.1 and M05.2, the AAMR remained similar at 0.2 per 100,000 (95% CI: 0.1–0.2), compared to the primary estimate of 0.2 per 100,000. The number of deaths decreased from 10,171 to 9,562, resulting in a slightly wider confidence interval. These findings suggest that the overall mortality estimates were not driven by these specific RA subtypes.

## DISCUSSION

In this nationwide analysis, RA-ILD mortality declined significantly over the past two decades, with the most pronounced improvement occurring between 2014 and 2017. This improvement may reflect advances in RA management, particularly the introduction and wider use of biologic and targeted synthetic DMARDs, alongside greater recognition and earlier diagnosis of ILD.^6,7^

Olson et al. (1988–2004) reported that, among 162,032 rheumatoid arthritis (RA)-associated deaths, 10,725 (6.6%) were attributed to RA-ILD.^8^ While overall age-adjusted mortality rates for RA declined, RA-ILD-associated mortality rates increased, particularly among older individuals. Women consistently had higher RA-ILD mortality than men, with an average of 2.9 per 1,000,000 population in women versus 1.8 per 1,000,000 in men. Similarly, our findings indicate that women constituted a larger proportion of RA-ILD deaths (63.5%, n=6,463) and had higher age-adjusted mortality rates than men (0.2 vs. 0.1), aligning with Olson et al.’s observation of higher RA-ILD mortality among women.

Jeganathan et al. (2005–2018) reported that RA-ILD-related mortality rates remained largely stable across both sexes, with women exhibiting slightly higher age-adjusted mortality than men (2.9 vs. 2.1 per 1,000,000), despite overall RA mortality declining in both sexes.^9^ In contrast, our analysis (1999–2020) demonstrates a more dynamic pattern: women accounted for 63.5% of RA-ILD deaths and had higher age-adjusted mortality rates than men (0.2 vs. 0.1). Specifically, women experienced a gradual, non-significant decline from 1999–2017 followed by a significant reduction between 2017–2020, whereas men showed an initial decline, a sharp drop from 2007–2010, and a modest, non-significant increase thereafter. While both studies consistently show higher mortality in women, our findings reveal evolving temporal trends for RA-ILD mortality not captured in the relatively stable patterns reported by Jeganathan et al.

Both studies reveal significant racial and ethnic disparities in RA-ILD mortality. Jeganathan et al. (2005–2018) identified the highest mortality rates among Native Americans, followed by Hispanics and Whites, while Black and Asian individuals had lower rates. Our analysis (1999–2020) corroborated these findings, noting American Indian/Alaska Native individuals as having the highest age-adjusted mortality rates, with non-Hispanic Black and Asian/Pacific Islanders at the lowest. Notably, while Jeganathan et al. reported higher mortality in Hispanics compared to Whites, our study found a non-significant decline in Hispanic mortality over time. Our study extends literature through 2020, capturing the sharp 2014–2017 decline and the modest post-2017 rise, a pattern not previously reported. The decline may reflect broader use of biologics, adoption of treat-to-target strategies, and earlier detection of ILD with high-resolution CT, while the recent uptick may relate to coding shifts or early impacts of the COVID-19 pandemic. Ongoing racial disparities, particularly among American Indian and Alaska Native populations, likely reflect structural barriers to care, including restricted Access to rheumatology and pulmonology services, higher prevalence of comorbid conditions, and broader systemic inequities within healthcare delivery.

Significantly, in contrast to earlier studies,^8,9,10^ our study provides a comprehensive geographic and rural urban comparisons, uncovering unique temporal trends and state-level variations, thereby providing a more detailed and granular perspective on RA-ILD mortality trends, which may facilitate targeted resource distribution. This aspect constitutes a principal strength of our research, emphasising enduring disparities and the progression of population-specific patterns in RA-ILD mortality.

Recent publications in the Mediterranean Journal of Rheumatology, including the multicentre Greek study by Karageorgas et al.,^11^ highlighted the limited availability of comprehensive mortality data for RA-ILD and emphasised the need for broader, systematic approaches to characterise disease burden and outcomes in Mediterranean populations. Our nationwide analysis, although conducted in North America, directly addresses this gap by demonstrating how large scale, standardised mortality surveillance can identify temporal trends, geographic variation, and population-level disparities in RA-ILD outcomes. The observed post 2014 mortality decline underscores the potential impact of earlier ILD screening and contemporary RA management, while persistent regional disparities in our data highlight that therapeutic advances must be accompanied by equitable access to specialised care, an important consideration for Mediterranean countries facing urban rural and health system heterogeneity.

## LIMITATIONS

This study has several limitations. Using multiple cause-of-death data may include deaths influenced by conditions other than RA-ILD, and death certificate coding carries a risk of misclassification or underreporting despite standardised ICD-10 use. Additionally, the database lacks clinical details such as disease duration, treatment exposures, and comorbidities, limiting exploration of underlying drivers of mortality. Nonetheless, the large national sample, extended study period, and stratified analyses provide a robust assessment of population-level RA-ILD mortality trends.

## CONCLUSIONS

RA-ILD mortality in the United States has declined over the past two decades, with notable improvements among women and persistently higher risk in American Indian and Alaska Native populations. These findings highlight the progress achieved through modern RA management and earlier ILD detection, while highlighting the need for targeted strategies to reduce disparities and sustain long-term gains in survival.

## Data Availability

The data used in this study are publicly available through the Centres for Disease Control and Prevention (CDC) WONDER Multiple Cause of Death database (https://wonder.cdc.gov/) and can be accessed without restriction.

## EDITORIAL AND AI ASSISTANCE

No professional editing services or editing agencies were used in the preparation of this manuscript. No part of this manuscript was drafted or edited using artificial intelligence based tools. All contents were written, reviewed, and approved by the authors. The authors take full responsibility for the integrity, originality, and accuracy of the manuscript.

## PRIOR PRESENTATION

Portions of this work were previously presented in abstract form at the ACR Convergence 2025 Annual Meeting.

Malik F, Ahmed J, Shah M, Uttam R, Fahimuddin M. Gender, Racial, and Geographic Trends in Mortality from Interstitial Lung Diseases Among Patients With Rheumatoid Arthritis in the U.S. Population, 1999–2020 [abstract]. Arthritis Rheumatol 2025;77(suppl 9). DOI unavailable.
